# The Daily Relation between Parental Rejection and Emotional Eating in Youngsters: A Diary Study

**DOI:** 10.3389/fpsyg.2017.00691

**Published:** 2017-05-11

**Authors:** Julie Vandewalle, Elien Mabbe, Taaike Debeuf, Caroline Braet, Ellen Moens

**Affiliations:** Department of Developmental, Personality and Social Psychology, Ghent UniversityGhent, Belgium

**Keywords:** parental rejection, emotional eating, adolescence

## Abstract

**KEY POINTS**

Cross-sectional survey studies have demonstrated significant associations between parental rejection and peer rejection on the one hand and disturbed eating in youngsters, like emotional eating, on the other hand. In this study, we wanted to expand our knowledge on these relationships by investigating the daily fluctuations in these variables. Youngsters completed a 7-day diary to assess daily parental rejection, peer rejection and emotional eating. Using multilevel analyses, our results showed that daily variations in parental rejection were related to daily variations in emotional eating of the youngsters. This highlights the importance of addressing the parent-child relationship in interventions for emotional eating in youngsters.

**Background:** This study investigated the daily relation between parental rejection and peer rejection on the one hand and emotional eating in youngsters on the other hand.

**Methods:** Participants (*N* = 55) between the ages of 11 and 15 years completed a 7-day diary. A multilevel design was used to examine day-to-day within-person relationships between parental and peer rejection (measured by CHS) and emotional eating (measured by DEBQ-C) of youngsters.

**Results:** The results showed that daily variations in parental rejection were related to daily variations in emotional eating of the youngsters. Daily peer rejection was only marginally significantly related to the emotional eating of the youngsters.

**Conclusions:** These results indicate that especially parental rejection, and to a lesser extent peer rejection, are associated with the emotional eating of youngsters. The findings highlight the importance of addressing the parent-child relationship in interventions for emotional eating in youngsters.

## Introduction

Emotional eating is defined as “the tendency to overeat in response to negative emotions such as anxiety or irritability” (van Strien et al., 2007, p. 106). Overeating can imply eating beyond the satiation point as well as eating in the absence of hunger. Emotional eating is characterized by eating comfort food, which is highly palatable food rich in sugar and fat, like desserts and fast food (Gibson, 2012). Thus, emotional eating might contribute to an unhealthy lifestyle and cause overweight or obesity over time (e.g., Braet et al., 2008; Bryant et al., 2008). In addition, the longitudinal study of Stice et al. (2002) demonstrated that emotional eating is an important predictor of binge eating. Binge eating is defined as eating large amounts of food in short periods of time, while losing control over eating is experienced. Binge eating is a key symptom of the mental disorders Binge Eating Disorder (BED) and Bulimia Nervosa (BN; American Psychiatric Association, 2013).

Emotional eating was first studied by Braet and Van Strien (1997), using parent reports. They found a good reliability and internal and external validity for the Dutch Eating Behavior Questionnaire- parent version (DEBQ; van Strien et al., 1986), as demonstrated by the significant positive correlations with energy-, fibers-, protein-, fat-, and sugar- intake/day. In a following study, Braet et al. (2007) compared the DEBQ- parent version (van Strien et al., 1986) and the DEBQ- child version (Braet et al., 2008). They found positive correlations between the two questionnaires, although they also found differences. Braet et al. (2007) recommend to also measure the unique perspective of the child. Studies indicate that emotional eating is already present in children and adolescents (e.g., Nguyen-Michel et al., 2007; Blissett et al., 2010). Braet and Beyers (2009) showed in different studies with children a prevalence of 40% emotional eating in community samples and more than 50% in obese samples. A study of Shapiro et al. (2007) showed that in their sample, 63% of children, aged between 5 and 13 years, answered affirmative when they were asked “Do you ever eat because you feel bad, sad, bored or any other mood.” In addition, Farrow et al. (2015) examined emotional eating in a longitudinal- experimental study by presenting snacks to satiated children and calculating their energy intake afterwards. The study showed the presence of emotional eating in children between the ages of 5 and 7 (Farrow et al., 2015). Using the DEBQ, Braet et al. (2008) found that in overweight 7–13 year old children, 10.5 % reported emotional eating. Michels et al. (2012) also showed in a longitudinal study the positive association between stress and emotional eating in 5–12 years old children. However, other research suggests that the prevalence of emotional eating is rather low in children, but increases in adolescence (e.g., Wardle et al., 2001; van Strien and Oosterveld, 2008). Further, research in patients with BED showed that, on average, the onset of binge eating occurs in adolescence, suggesting that this is a critical period in the development of binge eating (Spurrell et al., 1997). Since emotional eating is an important predictor of binge eating, early adolescence may be a crucial period for preventive efforts.

Emotional eating is still subject of investigation. Both questionnaire and experimental studies try to map the antecedents of emotional eating (Lindel et al., 2003; Goldschmidt et al., 2014a). Studies investigating these antecedents indicate that particularly incidents that elicit low- to moderately intense negative emotions, like daily hassles, trigger emotional eating (e.g., Macht, 2008). A study of O'Connor et al. (2003) revealed that daily hassles comprise many different events: ego-threatening, interpersonal, work-related hassles and physical stressors. The interpersonal model states that hassles related to interpersonal problems are a prominent predictor of disturbed eating behavior (Sullivan, 1953; Tanofsky-Kraff et al., 2007). According to this model, interpersonal problems elicit negative affect, which triggers disturbed eating behavior in an attempt to regulate the negative affect (Wilfley et al., 1997). Support for this model has been provided by research on binge eating in adults (e.g., Goldschmidt et al., 2014b), but has not been investigated for emotional eating.

Also other stressors, like parental control, which is associated with feelings of rejection by the child (Rohner, 1985), can influence the food intake of children. Research of Farrow et al. (2015) showed that 3–5 year-old children, whose parents controlled their food intake too much, subsequently developed an emotional eating style at the age of 5–7. Research on emotional eating in youngsters has demonstrated the importance of parental rejection (e.g., Vandewalle et al., 2014). Parental rejection is characterized by a lack of parental warmth and acceptance and/or the presence of physically and psychologically hurtful behaviors toward the child (Khaleque and Rohner, 2012). The association between parental rejection and emotional eating in youngsters has been confirmed in a clinical survey study in obese youngsters (Vandewalle et al., 2014) as well as in community-based survey studies (Schuetzmann et al., 2008; Vandewalle et al., 2016). Moreover, the results suggest that maternal rejection is more clearly linked to youngster's emotional eating than paternal rejection. All studies were cross-sectional except the study of Vandewalle et al. (2016), which included two time points (*M* = 71 days between time points). However, the results of this study did not show relations between changes in maternal rejection and changes in youngster's emotional eating. This may be explained by the large stability of maternal rejection found in this study. Although this study suggests that, in general, parental rejection is rather stable over longer times, based on attachment literature, day-to-day fluctuations in parental rejection probably do occur (Hazan and Shaver, 1987; Weinfield et al., 2000). Based on the interpersonal model, parental rejection may be related to day-to-day fluctuations in emotional eating. Therefore, additional research assessing day-to-day fluctuations in parental rejection and emotional eating may perhaps provide further insight into the hypothesized relationship between parental rejection and emotional eating in youngsters.

Peers are, next to the parents, the most important attachment figures for adolescents. Both parent- and peer acceptance are related to adjustment in adolescence, while parent- and peer rejection are associated with maladjustment, both emotional and behavioral (Rothbaum and Weisz, 1994; Hartup, 1996; Rohner and Britner, 2002; Sentse et al., 2010). Thus, next to parental rejection, peer rejection might also be an important antecedent of emotional eating in youngsters. Adolescence is characterized by increased importance of peer relationships, greater sensitivity to peer rejection and more psychological problems associated with peer rejection (Masten et al., 2009). Research in adolescents has found significant relationships between peer rejection and disturbed eating behavior, like binge eating (e.g., Gardner et al., 2007; Schutz and Paxton, 2007). However, to our knowledge, no studies have investigated the relationship between peer rejection and emotional eating in youngsters. As peer rejection may vary from day to day, a daily diary design may be an ideal methodology to explore the relationship between peer rejection and emotional eating.

### Aim of the present study

The present study aimed to examine the day-to-day within-person associations between parental and peer rejection and the emotional eating behavior of youngsters over 7 days, using a daily diary design. We hypothesized that (1) on between person level parental rejection and peer rejection will both be positively associated with emotional eating of youngsters, and (2) on within person level daily parental—and daily peer rejection will both be positively associated with daily emotional eating of youngsters.

## Methods

### Participants

Participants were enrolled in a larger study (Generation 2020) conducted at school, which investigated the school readiness of pupils. Parents and children were asked in a letter to indicate if they were interested to participate in a second part of the study, which would take place at home. If parents and youngster agreed, the family was contacted by telephone and information about the study was provided. Eighty-six families were contacted of which 59 families agreed to participate. Of these 59 participants, four participants were removed from the dataset because four or more of the seven daily entries were not completed or invalid (i.e., submitting data about multiple days in a single day). The final sample of 55 youngsters (49.1% boys and 50.9% girls) had a mean age of 12.36 (*SD* = 0.87) with a range of 11–15 years. The youngsters were measured and weighed by the researcher during a home visit. The BMI was calculated as weight (in kg)/height (in m^2^). We used the adjusted BMI ((actual BMI/percentile 50 of BMI for age and gender) × 100). This method allowed us to compare the BMI of children of different ages and gender. The 50th percentiles of the BMI for age and gender were based on normative data in a Flemish sample (Roelants et al., 2009). Adjusted BMI below 85% is classified as underweight, above 120% is classified as overweight and above 140% is classified as obese (Van Winckel and van Mil, 2001). The participants had a mean adjusted BMI of 102.86% (*SD* = 18.42) with a range of 76.66–153.28%. The familial socioeconomic situation was calculated using the Hollingshead Index of Social Position. The mothers of the youngsters were asked to fill in the education and occupation of both parents, which was then used to classify the families in one of five social position indexes. We recoded the five social position indexes into three social classes (high = upper and upper-middle, middle and low = lower-middle and lower). Most of the families (65.5%) were situated in the middle class, 27.3% in the high class and 7.3% in the lower class. The mothers were also asked to fill in the domestic situation of the youngster. The majority of the youngsters (92.7%) lived with both parents (intact family or co-parenting), two youngsters (3.6%) lived with mother and stepparent and two youngsters (3.6%) lived alone with their mother.

### Procedure

Trained psychology students visited the participants at home. First, the informed consent of both youngster and mother was obtained. The youngster and mother were then asked to fill in some questionnaires, which fall out of the scope of this study. Then, the youngster was weighed and measured by the psychology student. Next, the psychology student illustrated the use of the online diary. The youngsters were asked to visit the study website, log in with their unique code, provided by the researcher, and complete a test page as exercise. Youngsters were then instructed to complete the online diary questionnaires at the end of each day, for a period of seven consecutive days, starting the day after the home visit. Seven days is a common timeframe in diary studies, as it reduces participants fatigue and minimizes dropout (e.g., Almeida et al., 2002; Bolger et al., 2003). Participants answered the same questions each day and were asked to think about each item as they had experienced it that day. To help them remember to complete the diary each night, the youngsters were offered a glow in the dark bracelet. Furthermore, youngsters were given a paper version diary, so it was possible for them to write some things down during the day, which may help them to more accurately complete the online diary questionnaires at night. Afterwards, the researcher checked each day if the participants had completed the diary questionnaires, via an online administrator tool. If participants had failed to complete the diary questionnaires, an email was send the next day as a reminder. Via this email participants were instructed not to make up for the lost day, but instead complete the diary about the present day and continue until seven days were reached. As parental and peer rejection and emotional eating may differ between weekdays and weekend days, a comment was added that the diary should still be completed in 5 weekdays and 2 weekend days and that skipping other days was allowed in order to meet this requirement. If the next day participants still had not completed the diary questionnaires, a telephone call was made to offer assistance and further instructions. Participants were given a small voucher (e.g., shopping gift card) as reimbursement after their 7-day diary was completed. The study was approved by the Ethical Committee of Ghent University.

### Measures

#### Daily parental rejection and daily peer rejection

A shortened version of the Children's Daily Hassles Scale (CHS; Varni et al., 1996) was used to assess parental rejection and peer rejection. Each item of the original scale was examined on face validity. Regarding parental rejection, three items were retained from the original 25 items (“Your mother or father was mad at you for getting bad school results,” “Your mother or father did not have enough time for you,” “Your mother or father forgot to do something they had promised you”). In addition, three items were retained for peer rejection (“Children at school bullied or teased you,” “Your best friend no longer wanted to be your friend” “When children picked groups, you were picked as one of the last persons”). Participants were first asked to indicate if the rejection occurred that day. If the rejection had occurred, participants had to rate whether they felt “not bad” (1 point), “sort of bad” (2 points) or “very bad” (3 points) as a result of this rejection. The intensity score (severity sum of the rejection that occurred that day) was calculated for parental rejection as well as for peer rejection.

#### Daily emotional eating

An adapted version of The Dutch Eating Behavior Questionnaire - child version (DEBQ; van Strien et al., 1986; Braet et al., 2008) was used to assess the daily emotional eating behavior of the youngster. The DEBQ consists of 33 items, assessing, via self- report, the presence of three types of disturbed eating behavior: restrained eating, external eating, and emotional eating. Only an adapted version of the subscale emotional eating was assessed in this study. Original items are formulated as specific eating behaviors and have to be rated on their frequency of occurrence on a five-point Likert scale from 1 = never to 5 = very often (13 items; e.g., “If you're angry, do you have the desire to eat?”). In the current study, items were adapted so that participants were asked if they had experienced the emotion that day and if they then felt like eating (e.g., “Were you angry today and did you then have the desire to eat?”). Items were rated on a fivepoint Likert scale from 1 = not at all to 5 = very much. Studies have demonstrated the usefulness of the Dutch version of the DEBQ in children and adolescents between the age of 7 and 17 years (Braet et al., 2003, 2004). Research showed a stable factor structure, satisfying internal consistency reliability and good test–retest reliability (Braet et al., 2008). Furthermore, research showed a good external validity for the DEBQ in children (Ricciardelli and McCabe, 2001).

### Plan of analysis

In this diary study, several variables were repeatedly measured on 7 days (i.e., Level 1), which were nested within 55 participants (i.e., Level 2). To take into account between- and within-person differences, multilevel analyses were conducted with the statistical software package MLwiN 2.16 (Rasbash et al., 2015). Predictor variables at Level 1, reflecting within-participant predictors, were group-mean centered (i.e., centered around the participant's mean), whereas the continuous predictors at Level 2, reflecting between-participant predictors, were centered around the grand mean.

First, intercept-only models (null Model) were estimated to examine whether there was significant daily variability in emotional eating, parental rejection and peer rejection. These models do not explain any variance, but decompose the total amount of variance into the proportion of variance that is due to the between-person and within-person variation. This proportion is reflected in the intraclass correlation (ICC). In a next step, daily peer rejection and daily parental rejection were simultaneously entered in the Model without the background variables (Model 1). Subsequently, daily peer rejection (i.e., Level 1) and several background variables at Level 2 were entered simultaneously in a first Model as predictors of daily emotional eating (Model 2). Age, gender and adjusted BMI were included as background variables, as previous research has shown that these variables may have an effect on the emotional eating of youngsters (Braet et al., 2008). Lastly, daily parental rejection (i.e., Level 1) and the background variables (age, gender, and adjusted BMI) at Level 2 were entered simultaneously in a second Model as predictors of daily emotional eating (Model 3).

#### Results descriptive statistics and preliminary analyses

Table 1 shows correlations, means, and standard deviations of the diary variables and background variables age and adjusted BMI.

**Table 1:** **Descriptive statistics and correlations between dispositional and daily variables**.

	***M***	***SD***	**Range**	**1**	**2**	**3**	**4**
**BETWEEN PERSON LEVEL**							
1. Daily Peer Rejection	0.06 (0.04)^a^	0.22	0–3.00	–			
2. Daily Parental Rejection	0.30 (0.13)^a^	0.55	0–6.00	−0.11	–		
3. Daily Emotional Eating	14.59	2.90	13–42	0.28^*^	0.52^**^	–	
4. Age	12.36	0.87	11–15	0.24^*^	−0.11	0.04	–
5. Adjusted BMI	102.36	18.42	76.66–153.28	−0.10	−0.05	−0.03	0.15
**WITHIN PERSON LEVEL**							
1. Daily Peer Rejection	0.06	0.22	0–3.00	–			
2. Daily Parental Rejection	0.30	0.55	0–6.00	−0.01	–		
3. Daily Emotional Eating	14.59	2.90	13–42	0.10	0.19^**^		

* p < 0.05;

** *p < 0.01*.

a *Mean frequency of rejections*.

The range for completing the 7 days of the diary was between seven and 19 days. Of the 55 participants, 29 completed the diary in 7 days, and 26 participants needed more time. Of those 26 participants, 18 completed the diary in 8 or 9 days, two in 10 days, four in 11 days, one in 13 days and one participant needed 19 days.

### Primary analyses

#### Day-to-day variability in the study variables

Regarding the outcome variable, the ICC value indicated that 52% of the variance in emotional eating reflects between-person differences. With regard to daily parental and daily peer rejection, the ICC values indicated that, respectively, 28 and 31% of the variance reflect between person differences. This means that most of the variance (i.e., around 50% and more) for all day-level variables is situated at the within-person level, although that part of this within-person variance also reflects measurement error.

#### Daily parental rejection, peer rejection, and emotional eating

Table 2 presents the findings for daily emotional eating. In the Model without the background variables (Model 1), daily parental rejection had a significant positive association with daily emotional eating. Daily peer rejection had a marginally significant positive association with daily emotional eating. As can be noticed in Model 2, daily peer rejection had a marginally significant positive association with daily emotional eating. Model 3 showed that the frequency of daily parental rejection had a significant positive association with daily emotional eating. Table 2 also shows that there was no significant effect of age, gender or BMI on emotional eating, neither in Model 2 nor Model 3.

**Table 2:** **Daily emotional eating as a function of daily peer rejection and daily parental rejection and person level variables**.

	**Null model**	**Model 1**	**Model 2**	**Model 3**
**Fixed effects**				
Overall Intercept	14.58 (0.39)^***^	14.52 (0.39)^***^	14.43 (0.55)^***^	14.44 (0.55)^***^
**Day level**				
Daily Peer Rejection		1.01 (0.53)^†^	0.99 (0.54)^†^	
Daily Parental Rejection		0.66 (0.19)^***^		0.66 (0.19)^***^
**PERSON LEVEL**				
Adjusted BMI			−0.01 (.02)	−0.01 (0.02)
Gender			0.29 (0.77)	0.29 (0.77)
Age			0.13 (0.45)	0.13 (0.45)
**Random effects**				
u_0_	7.19 (1.56)^***^	7.24 (1.56)^***^	7.16 (1.55)^***^	7.19 (1.55)^***^
e_0_	6.487 (0.52)^***^	6.186 (0.50)	6.42 (0.51)^***^	6.26 (0.50)^***^
−2^*^loglikelihood	1844.429	1829.595	1840.787	1832.901

*Random effects at the between person level: u_0_ (amount of between-person variation)*.

Random effects at the within-person level: e_0_ (amount of within-person variation)

† *p < 0.1*,

*** *p < 0.001*.

#### Weekend vs. week variables

Analyses on daily emotional eating, daily parental rejection and daily peer rejection on the weekend vs. week variable showed that the weekend vs. week variable was not significantly associated with daily emotional eating (*B* = −0.17; *SE* = 0.30; *p* = ns) nor with daily parental rejection (*B* = 0.04; *SE* = 0.09; *p* = ns). The weekend vs. week variable was significantly associated with daily peer rejection (*B* = 0.09; *SE* = 0.30; *p* < 0.01). This means that they perceived significantly less peer rejection during the weekend, presumably because there was less contact with peers compared to the week/school days.

## Discussion

This study aimed to examine the day-to-day within-person associations between parental and peer rejection and emotional eating of youngsters over seven days. Firstly, results showed significant daily variability in parental rejection, peer rejection and emotional eating, making it possible to examine the relationships between these variables. As predicted, at the between person level, parental rejection was positively related to the emotional eating of the youngsters. This corresponds with the cross-sectional results of Vandewalle et al. (2014) and Schuetzmann et al. (2008) and suggests, for the first time using a naturalistic daily diary design, that parental rejection is associated with emotional eating in youngsters. Secondly, in contrast to our hypothesis, the association between daily peer rejection and emotional eating was marginally significant. This corresponds with the study of Masten et al. (2009) and Gardner et al. (2007) who found positive relationships between peer rejection and respectively psychological problems and binge eating in adolescents. Thirdly, within one person, daily parental rejection was also found to be positively related to daily emotional eating: days characterized by higher levels of parental rejection went along with higher emotional eating. These results suggests that especially parental rejection is associated with the emotional eating of youngsters. Scholte and Van Aken (2006) state that, although peer relationships become increasingly important in adolescence, the relationship between parent and adolescent is still significant in adolescents' lives. For example, research has shown that family support, and not peer support, has a protective influence on the development of depression in adolescents (McFarlane et al., 1994, 1995). Furthermore, longitudinal research suggests that peer rejection may be a consequence of the emotion regulation behavior of children, instead of an antecedent (e.g., Trentacosta and Shaw, 2009; Kim and Cicchetti, 2010). Children who have difficulty managing their negative emotions have more chance of becoming disruptive in social relationships, which may lead to lower peer acceptance and more peer rejection (Maszk et al., 1999). Furthermore, it should be noted that descriptive analysis of the study variables in our sample showed that youngsters reported less daily peer rejection than daily parental rejection. Additionally, the variance of daily peer rejection was smaller than the variance of daily parental rejection, which might explain the marginally significant relationship with emotional eating.

There are a few limitations to this study that require comment. Firstly, Bongers et al. (2013) state that people are generally rather poor at recalling their emotions, their eating behavior and the associations between the two. Thereby questioning the validity of emotional eating, this study tried to overcome this by using a daily diary method. Using event- or signal- contingent diary methods may fully eliminate this recall bias. However, these methods may have the disadvantage of being too burdensome for the participants, which may decrease the motivation and increase the drop-out (Powell, 2015). To make our design less burdensome, we gave the participants the choice to skip some days in the reporting, but this might have influenced our results. It might be possible that participants skipped the days when the amount of rejection and/ or emotional eating was higher. In addition, using multiple assessment moments throughout the day may influence participants' eating behavior during that day. Furthermore, recent event- or signal- contingent diary studies utilize a palm top computer or smartphone app to signal the participants at the end of each interval or to facilitate event-contingent assessment. Considering the sample's age, it should be taken into account that some schools do not allow the use of electronical devices.

Nonetheless, it would be interesting if future research would include complementary measures of emotional eating. Also interesting would be to assess further stress during the day, which we did not consider in this study. Additionally, it would be interesting to include data from other informants, like the parents. In this study, only youngsters' perception was assessed, which might be affected by the high level of abstraction that is needed to complete the diary- questions. Including multi-method and multi-informant data would offer more objective evidence of the relationship between parental rejection and the eating behavior of youngsters. Secondly, these study results are preliminary, as the use of a day level retrospective report makes causal inferencing impossible. We can only make conclusions in an associative way but not in terms of causality. Thirdly, parental rejection might be viewed as a feature of the broader family climate, given the stability found in longitudinal research (Vandewalle et al., 2016). A clear measure of parental rejection on a day-to-day level might be more appropriate. Furthermore, as we preserved the original items of the CHS, we did not distinguish between maternal and paternal rejection, what could be seen as a limitation in this study. Considering research suggests that maternal rejection is more clearly linked to youngster's emotional eating than paternal rejection, it would be interesting in future research to investigate the influence of daily maternal and paternal rejection on daily emotional eating of youngsters separately (Vandewalle et al., 2014, 2016). Also interesting would be to investigate the distinction between rejection of a primary caregiver and rejection of secondary caregivers. By doing research on these different “sources” (namely maternal; paternal and no primary caregiver) of rejection, a lot of new, important and (clinical) relevant information could be found (e.g., child gender by parental source interactions,). Another consideration for further research is to include the measurement of existing eating disorders. It might be possible that eating disorders have an influence on the amount of emotional eating.

## Conclusions

To conclude, the results of this study demonstrated the relationship between daily parental rejection and daily emotional eating in youngsters. Therefore, it might be advisable to assess the parent-child relationship, and if mandatory, improve the parent-child relationship in the treatment of emotional eating in youngsters. Interpersonal psychotherapy, based on the interpersonal model of binge eating, focuses on helping the patient develop strategies for improving interpersonal functioning (Tanofsky-Kraff et al., 2007; Cassidy et al., 2013) and has been proven to be effective in reducing binge eating in adults in the short- and long-term (Kass et al., 2013). Furthermore, preliminary evidence from a pilot study suggests that this therapy may also reduce loss of control over eating and lead to a smaller BMI increase over 1 year in adolescent girls (Tanofsky-Kraff et al., 2010). It would be fruitful if future research would test the effectiveness of interpersonal psychotherapy on the parent-child relationship and the emotional eating in youngsters.

## Author contributions

All authors listed, have made substantial, direct and intellectual contribution to the work, and approved it for publication.

### Conflict of interest statement

The authors declare that the research was conducted in the absence of any commercial or financial relationships that could be construed as a potential conflict of interest.
